# Physician Voting Rates in the 2020 and 2022 US Elections

**DOI:** 10.1001/jamahealthforum.2024.5443

**Published:** 2025-02-14

**Authors:** Julianna Pacheco, Nathan K. Micatka, Caroline Tolbert

**Affiliations:** 1 Department of Political Science, The University of Iowa, Iowa City

## Abstract

This cross-sectional study compares the actual voting rates between physicians and the general adult population in the US.

## Introduction

US physicians are leaders in health care policy and are critical for public health yet may be less likely to vote than the general population.^1^ Most studies of physician voting have used surveys with self-reported voting data. For example, a 2021 study using the 2004 to 2018 Current Population Survey (CPS) from the US Census Bureau found physicians were 12% less likely to vote than the general public.^2^

Self-reported voting rate is often higher than the actual voting rate because of social desirability bias, when survey respondents answer questions in a manner others will view favorably. The CPS tends to overreport turnout by Asian, Black, and Hispanic individuals and underreport turnout by non-Hispanic White people.^3^ In this study, we used administrative data from state voter records to evaluate physician voting rates in the 2020 presidential and 2022 midterm elections.

## Methods

This cross-sectional study used administrative data from a random 1% sample of the 2023 US adult population from Catalist, a private company that maintains a national voter registration database appended with additional variables from commercial, governmental, and other administrative sources. Individual-level data included voting status in 2020 and 2022, voting history from 2016 and 2018, demographics, and physician licensure (doctor of medicine; doctor of osteopathic medicine) from state medical boards in 2019. The sample included registered and unregistered adults (aged ≥18 years), approximately 0.25% of whom were identified as licensed physicians, which reflects the estimated national figure of licensed physicians (approximately 0.30% of the general population). The University of Iowa Institutional Review Board deemed this study exempt from review and waived informed consent because deidentified administrative third-party data were used. We followed the STROBE reporting guideline.

Voting rates of physicians and other US adults were compared using multivariable logistic regression models. These models reported marginal effects of voting for physicians vs nonphysicians, controlling for voting history in 2016 and 2018; demographics, including age, education, and income; and Cost of Voting Index, a composite score of 33 state election laws from 1996 to 2016,^4^ with higher scores indicating more restrictive voting laws. State-level fixed effects were included to account for other state factors. Analyses were conducted using Stata SE, version 18 (StataCorp LLC).

## Results

The sample included 8913 physicians (3171 females [35.6%], 5742 males [64.4%]) and 3 607 654 nonphysicians (1 930 493 females [53.5%], 1 677 161 males [46.5%]). Compared with the general population, physicians were older (median [IQR] age, 51 [36-67] vs 57 [46-69] years), more likely to be males (46.5% vs 64.4%), and more likely to be non-Hispanic White individuals (70.7% vs 75.5%). In adjusted analyses, there was an association between being a physician and voting (Table). Model 3 showed that physicians’ adjusted voting probability in the 2020 presidential election was higher than nonphysicians’ (70.0% [95% CI, 67.7%-70.2%] vs 62.6% [95% CI, 62.5%-62.7%]). Similarly, in the 2022 midterm election, physicians’ adjusted voting probability was higher than nonphysicians’ (48.4% [95% CI, 47.3%-49.4%] vs 45.3% [95% CI, 45.2%-45.4%]).

**Table.** Logistic Regression Models of Voting in 2020 and 2022 US Elections Among Physicians and Nonphysicians^a^

Variable	Marginal effects (95% CI), percentage points					
	2020 Presidential election			2022 Midterm election		
	Model 1: adjusted turnout demographic (n = 2 475 577)	Model 2: adjusted turnout demographic and prior vote (n = 2 475 577)	Model 3: full turnout (n = 2 468 188)^b^	Model 4: adjusted turnout demographic (n = 2 475 577)	Model 5: adjusted turnout demographic and prior vote (n = 2 475 577)	Model 6: full turnout (n = 2 468 188)^b^
Voted in 2016	NA	0.450 (0.450 to 0.451)	0.413 (0.413 to 0.414)	NA	NA	NA
Voted in 2018	NA	NA	NA	NA	0.409 (0.409 to 0.409)	0.373 (0.373 to 0.374)
Physician, ever been	0.309 (0.292 to 0.327)	0.125 (0.112 to 0.138)	0.063 (0.050 to 0.076)	0.228 (0.215 to 0.242)	0.077 (0.067 to 0.088)	0.030 (0.020 to 0.041)
Age	0.003 (0.003 to 0.003)	−0.001 (−0.001 to −0.001)	−0.001 (−0.001 to −0.001)	0.005 (0.005 to 0.005)	0.001 (0.001 to 0.001)	0.001 (0.001 to 0.001)
Sex						
Female	0.020 (0.019 to 0.021)	0.001 (0.000 to 0.002)	0.008 (0.008 to 0.009)	−0.004 (−0.005 to −0.003)	−0.012 (−0.013 to −0.011)	0.001 (0.000 to 0.002)
Male	1 [Reference]	1 [Reference]	1 [Reference]	1 [Reference]	1 [Reference]	1 [Reference]
Race and ethnicity^c^						
Asian	−0.104 (−0.107 to −0.101)	−0.004 (−0.007 to −0.002)	−0.048 (−0.050 to −0.045)	−0.161 (−0.164 to −0.157)	−0.073 (−0.076 to −0.071)	−0.098 (−0.101 to −0.096)
Black	−0.124 (−0.126 to −0.122)	−0.074 (−0.076 to −0.073)	−0.034 (−0.036 to −0.032)	−0.164 (−0.166 to −0.162)	−0.118 (−0.119 to −0.116)	−0.062 (−0.063 to −0.060)
Hispanic	−0.168 (−0.170 to −0.167)	−0.07 (−0.071 to −0.068)	−0.052 (−0.054 to −0.050)	−0.226 (−0.228 to −0.224)	−0.132 (−0.133 to −0.130)	−0.103 (−0.105 to −0.101)
White, non- Hispanic	1 [Reference]	1 [Reference]	1 [Reference]	1 [Reference]	1 [Reference]	1 [Reference]
Other^d^	−0.111 (−0.114 to −0.108)	−0.036 (−0.039 to −0.033)	−0.039 (−0.042 to −0.036)	−0.133 (−0.136 to −0.129)	−0.069 (−0.072 to −0.066)	−0.062 (−0.065 to −0.059)
Income, 7 categories^e^	NA	NA	0.026 (0.025 to 0.027)	NA	NA	0.047 (0.047 to 0.048)
Probability of a bachelor’s degree, 0-100 scale^f^	NA	NA	0.002 (0.002 to 0.002)	NA	NA	0.000 (0.000 to 0.000)
Married	NA	NA	−0.013 (−0.014 to −0.012)	NA	NA	0.016 (0.014 to 0.017)
COVI	NA	NA	0.005 (0.002 to 0.007)	NA	NA	0.031 (0.029 to 0.034)

Abbreviations: COVI, Cost of Voting Index (scale: 0-100, with higher scores indicating more restrictive voting laws); NA, not applicable.

^a^ Calculated using Catalist data, representing a 1% random sample of the US population.

^b^ State-level fixed effects were used in model 3 and model 6 by including a binary variable for each state, with Alabama omitted as the reference.

^c^ Race and ethnicity were either self-reported by individuals to their state through voter registration or modeled by Catalist to report the most likely races and ethnicities of individuals based on large sample surveys and industry data. Race and ethnicity were assessed in this study due to their association with electoral participation.

^d^ Other category included American Indian and Alaska Native and unknown.

^e^ Income was provided by credit bureau reports and included in Catalist data. The 7 categories included <$20 000, $20 000-$30 000, $30 000-$50 000, $50 000-$75 000, $75 000-$100 000, $100 000-$150 000, and >$150 000.

^f^ Education was a modeled variable from Catalist. The probability that an individual has a college degree was measured using a 0-100 scale.

The Figure shows the probabilities of voting for physicians by state from model 3 in the Table. The highest probabilities of physician turnouts were in Utah (95.7%), Wyoming (96.7%), and North Dakota (98.6%), with the lowest being in Arkansas (76.0%).

**Figure.**
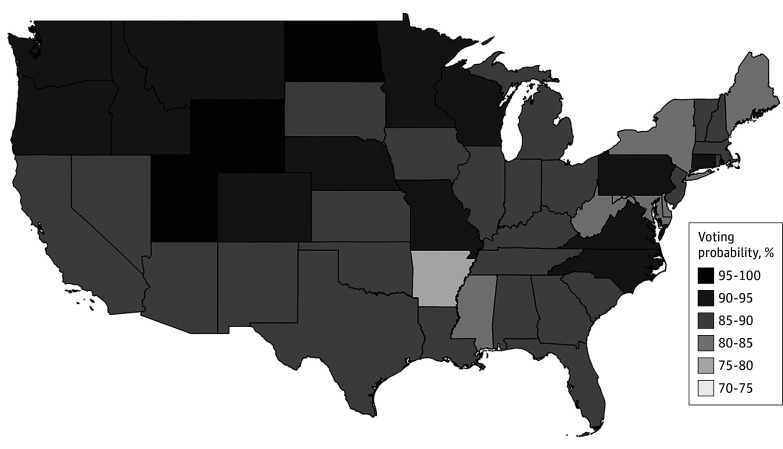
Estimated Probabilities of Physicians Voting in the 2020 US Presidential Election Probabilities are based on model 3.

## Discussion

Using licensure data from state medical boards to identify physicians and linking these data with state voter files, we found that physicians voted at significantly higher adjusted rates than other adults in the 2020 presidential and 2022 midterm elections in the US. This finding is consistent with results of previous work from 2020.^5^

Study limitations include the limited time frame and the inability to validate the linkage of physician license and voting records conducted by Catalist. Given their high voting rates, physicians may be well suited to advise patients to register and vote through organized mobilization efforts.^6^

## Notes

Supplement.
Data Sharing Statement
