# Supplementary material for: Physician Voting Rates in the 2020 and 2022 US Elections
Source: JAMA Health Forum. 2025 Feb 14;6(2):e245443. doi: 10.1001/jamahealthforum.2024.5443 (PMC11829221; doi:10.1001/jamahealthforum.2024.5443)
Supplement: Supplement. — Data Sharing Statement [file jamahealthforum-e245443-s001.pdf]

Data Sharing Statement

Pacheco. Physician Voting Rates in the 2020 and 2022 US Elections. *JAMA Health Forum*. Published February 14, 2025. doi:10.1001/jamahealthforum.2024.5443

Data
Data available: Yes
Data types: Data (not involving human participants)
How to access data: The aggregate data can be available. Make requests to julianna-pacheco@uiowa.edu
When available: With publication

Supporting Documents
Document types: None

Additional Information
Who can access the data: Anyone requesting the data
Types of analyses: For any purpose
Mechanisms of data availability: After email request
Any additional restrictions: We cannot share individual level data because it is proprietary. Can only share aggregated state data.
